# Structural Basis for Functional Tetramerization of Lentiviral Integrase

**DOI:** 10.1371/journal.ppat.1000515

**Published:** 2009-07-17

**Authors:** Stephen Hare, Francesca Di Nunzio, Alfred Labeja, Jimin Wang, Alan Engelman, Peter Cherepanov

**Affiliations:** 1 Division of Medicine, St. Mary's Campus, Imperial College London, London, United Kingdom; 2 Department of Cancer Immunology and AIDS, Dana-Farber Cancer Institute, Boston, Massachusetts, United States of America; 3 Department of Molecular Biophysics and Biochemistry, Yale University, New Haven, Connecticut, United States of America; University of Geneva, Switzerland

## Abstract

Experimental evidence suggests that a tetramer of integrase (IN) is the protagonist of the concerted strand transfer reaction, whereby both ends of retroviral DNA are inserted into a host cell chromosome. Herein we present two crystal structures containing the N-terminal and the catalytic core domains of maedi-visna virus IN in complex with the IN binding domain of the common lentiviral integration co-factor LEDGF. The structures reveal that the dimer-of-dimers architecture of the IN tetramer is stabilized by swapping N-terminal domains between the inner pair of monomers poised to execute catalytic function. Comparison of four independent IN tetramers in our crystal structures elucidate the basis for the closure of the highly flexible dimer-dimer interface, allowing us to model how a pair of active sites become situated for concerted integration. Using a range of complementary approaches, we demonstrate that the dimer-dimer interface is essential for HIV-1 IN tetramerization, concerted integration in vitro, and virus infectivity. Our structures moreover highlight adaptable changes at the interfaces of individual IN dimers that allow divergent lentiviruses to utilize a highly-conserved, common integration co-factor.

## Author Summary

Integrase is the viral enzyme that orchestrates insertion of both ends of retroviral DNA into a host cell chromosome. This process, thought to require a tetramer of integrase, involves two concerted cutting/joining (transesterification) reactions that target a pair of phosphodiester bonds in chromosomal DNA, separated by ∼18 Å. Until now, the architecture of the integrase tetramer responsible for concerted integration has remained a mystery. We now report two crystal structures containing the N-terminal and catalytic core domains from a lentiviral integrase in complex with its co-factor LEDGF. Comparison of the structural arrangements observed in our crystals elucidates the details of the integrase tetramerization interface, reveals its dramatic flexibility and the mechanism by which a pair of active sites can be brought into close proximity. Taking advantage of the structural data, we generated a series of HIV-1 integrase mutants designed to disrupt or re-create its tetramerization interface. Biochemical and virus replication studies with these mutants strongly support the functional significance of the tetrameric architecture observed in the crystal structures. Our results provide important novel insights into the assembly of the functional integrase tetramer and will be invaluable for the ongoing efforts to model the retroviral pre-integration complex.

## Introduction

To establish productive infection, a retrovirus must insert the reverse-transcribed form of its genome into a host cell chromosome. This process critically depends on two reactions, 3′-processing and strand transfer, catalyzed by the viral enzyme integrase (IN) (reviewed in [1]). During 3′-procesing, IN endonucleolytically removes two or three nucleotides from the 3′-termini of viral DNA to expose 3′-OH groups of invariant CA dinucleotides. These are subsequently utilized in a pair of coordinated transesterification reactions, resulting in the insertion of both viral DNA termini across the major groove of chromosomal DNA. Integration is completed through the action of host DNA repair enzymes, which mediate the necessary joining of viral DNA 5′-ends, yielding a short duplication of target DNA sequence flanking the integrated provirus.

Retroviral INs have a characteristic three-domain organization, all containing N-terminal, catalytic core and C-terminal domains (NTD, CCD, CTD) (reviewed in [2]). The CCD contains the invariant D,D-35-E motif responsible for coordination of two Mg^2+^ ions within the active site and accounts for sequence-specific interactions with viral DNA [3],[4]. The positively-charged CTD is also implicated in DNA binding, likely accounting for sequence-independent interactions [5]. All three domains contribute to IN multimerization [6]–[8]. CCDs of divergent retroviral INs invariably crystallize as dimers, with isomorphous dimer interfaces [9]–[11]. Structures of the NTD and CTD have been solved both alone and as part of two-domain constructs involving the CCD by respective use of NMR and crystallography [12]–[15]. The NTD forms a three-helical bundle stabilized through coordination of a Zn^2+^ ion by the invariant HHCC motif. The CTD consists of a five-stranded β-barrel similar to Src homolgy 3 domains.

Although the structure of full-length retroviral IN remains elusive, its partial structures were instrumental in unraveling the mechanism of integration. The near-spherical CCD dimer cannot alone explain the concerted integration of two viral DNA ends. Indeed, the active sites, located on opposite sides of the dimeric CCD structure, are separated by ∼40 Å, while the distance between target scissile bonds in ideal B form DNA is close to 18 Å. A tetramer would be the minimal IN multimer to provide a pair of active sites with the expected spacing, and available experimental evidence suggests that the functional form of retroviral IN is indeed tetrameric [16]–[19]. An attractive model was derived from the crystal structure of a two-domain fragment of HIV-1 IN (IN_NTD+CCD_) [15]. Although lacking the CTD, this construct crystallized in tetrameric form, best described as a dimer-of-dimers, with the dimers interacting with each other predominantly via NTD-CCD contacts. This model was inviting because it showed some structural similarity to the synaptic complex of the related Tn5 transposase [20] and, while the ∼29 Å separation of active sites was too far to accommodate concerted integration, it seemed plausible that flexibility along the dimer-dimer interface could provide the necessary geometry.

For efficient integration, HIV-1 and other lentiviruses depend on lens epithelium derived growth factor (LEDGF) [21]–[23] (reviewed in [24]), a cellular chromatin-associated protein implicated in transcription regulation and apoptosis [25],[26]. LEDGF directly interacts with lentiviral IN proteins and is thought to tether the preintegration complex to chromatin for strand transfer [27]–[29]. The CCD of HIV IN is the main determinant for the interaction with LEDGF, although the NTD is required for high-affinity binding [28],[30]. Reciprocally, a small alpha-helical domain within the C-terminal portion of LEDGF is necessary and sufficient for the interaction with IN [31],[32]. Crystal structures of the integrase-binding domain (IBD) of LEDGF (LEDGF_IBD_) in complex with HIV-1 IN_CCD_ and HIV-2 IN_NTD+CCD_ have revealed molecular details of this interaction [30],[33].

Herein we present two new crystal structures containing the NTD and the CCD of maedi-visna virus (MVV) IN in complex with LEDGF_IBD_. In both structures, this highly divergent lentiviral IN is present in tetrameric forms, stabilized by swapping pairs of NTDs between interacting dimers. Comparison of four independent IN tetramers observed in our structures reveals variability of the dimer-dimer interface, which affords juxtaposition of a pair of active sites for concerted integration. Using a range of complementary functional assays, we show that the tetramerization interface is essential for IN function, both in vitro and in the context of viral replication.

## Results

### Crystal structures of the MVV IN_NTD+CCD_:LEDGF_IBD_ complex

To ascertain protein-protein interfaces involved in retroviral integration, we sought to determine crystal structures of divergent lentiviral INs. MVV IN presented an appealing target because it shares less than 30% overall sequence identity with its HIV-1 counterpart (Figure S1). Opportunely, sequence analysis of LEDGF cDNA isolated from sheep, a natural MVV host, confirmed that the amino acid sequence of its IBD is identical to that of the human ortholog. Bacterial co-expression of MVV IN_NTD+CCD_ (residues 1–219) with LEDGF_IBD_ yielded monodisperse preparations of the protein-protein complex without introducing solubilizing point mutations into the IN construct. The protein complex crystallized in two forms, referred to as crystal form (CF) 1 and CF2, and the resulting structures were refined to 3.28 and 2.64 Å, respectively (Table 1).

**Table 1:** Summary of crystallographic statistics.

	CF1	CF2
**Data collection:**		
Resolution range^a^ (Å)	40-3.28 (3.46-3.28)	40-2.64 (2.71-2.64)
R_merge_ a	11.0 (66.5)	10.2 (58.6)
Multiplicity^a^	3.8 (3.8)	3.4 (3.3)
I/σ_I_ a	11.2 (2.0)	8.1 (2.1)
Completeness^a^ (%)	99.6 (99.7)	99.5 (99.4)
**Refinement:**		
Resolution (Å)	40-3.28	40-2.64
Reflections work set	32,366	50,264
Reflections test set	1,718	2,827
R_work_ (%)	21.28	22.63
R_free_ (%)	25.51	25.30
No. protein atoms	12,786	8,625
No. ligand/ion atoms	6	43
No. water molecules	0	110
R.m.s. bonds (Å)	0.009	0.013
R.m.s. angles (°)	1.134	1.406
Ramachandran plot (%):		
Favored	93.6	96.6
Allowed	5.2	3.2
Outliers	1.2	0.2

a Data in parentheses represent highest resolution shell.

The asymmetric unit (ASU) of CF1 contains three IN dimers (chains A–F), each with a pair of associated LEDGF chains (G–L). The dimers interact with each other to form three independent dimer-dimer interfaces, such that the EF dimer interacts with the AB and CD dimers, and the CD dimer with the A′B′ dimer from another ASU (Figures S2A–S2C). The ASU of CF2 contains a pair of IN dimers that form a single tetramer with four associated LEDGF chains (Figure S2D). Although in most IN chains the loops connecting NTDs and CCDs are disordered, clear electron density was seen in chain B of CF1, allowing unambiguous assignment of all NTDs in this crystal form (Figure S2C). In CF2, where the NTD-CCD linkers are disordered for all monomers, unambiguous assignment of IN chain B and C NTDs (cyan and yellow in Figure S2D) was possible due to distance restraints: the shortest path to connect chain B Gln44 with chain C Ser55, while avoiding clashes with the rest of the model, would be well over 50 Å, a distance that cannot be covered by 10 amino acid residues.

### IN tetramerization is primarily mediated by intermolecular NTD-CCD interactions

**Figure 1:**
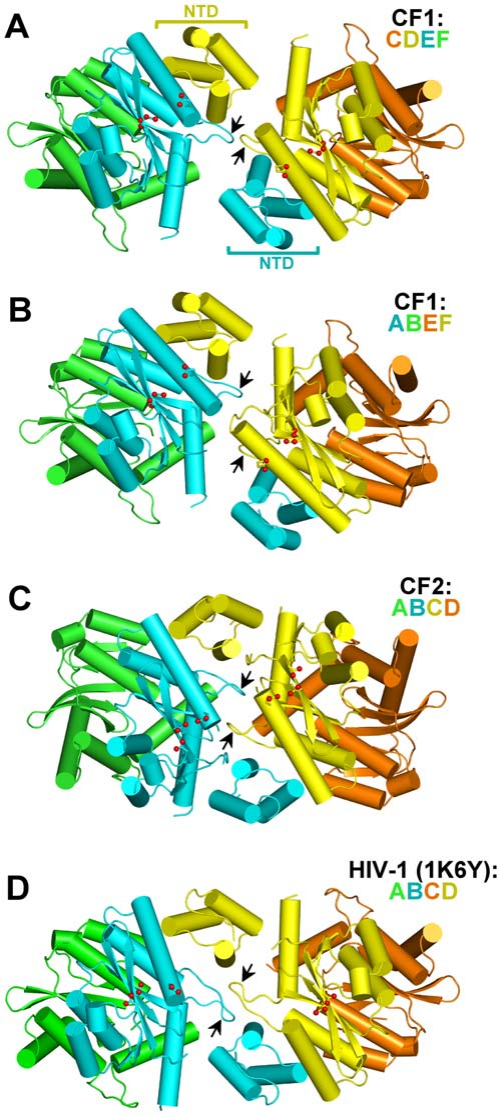
Observed lentiviral IN tetramers. MVV IN tetramers from CF1 and CF2 structures (A–C), compared to the HIV-1 IN tetramer from Wang et al. [15] (PDB ID 1k6y) (D). For clarity, the outer NTDs and LEDGF chains are omitted. The CF1/CDA′B′ tetramer, which is very similar to CF1/ABEF, is not shown. Protein chains, shown as cartoons, are color-coded as indicated; cylinders represent α helices. Catalytic triad residues (Asp66, Asp118 and Glu154 in MVV; Asp64, Asp116 and Glu152 in HIV-1) belonging to the inner monomers of each tetramer (cyan and yellow chains) are shown as sticks, the carboxylate oxygen atoms highlighted as red spheres. The black arrowheads indicate the CCD fingers of the inner monomers, which participate in tetramerization.

Collectively, CF1 and CF2 reveal four independent IN tetramers (Figure S2). Within each tetramer a pair of NTDs (henceforth referred to as inner NTDs) mediate stable dimer-dimer interactions. The remaining (outer) NTDs do not share a conserved role or position within the tetramers (Figure S2). The salient details of higher-order dimer-dimer interaction are shown for three of the four tetramers (CF1/IN chains CDEF, CF1/ABEF, and CF2/ABCD) in Figure 1A–1C, with LEDGF chains and outer NTDs omitted for clarity. The interface within the CF1/CDA′B′ tetramer is very similar to that in ABEF, and will therefore not be discussed separately. Within tetramers, the positions of the inner NTDs relative to the opposing CCD dimers are maintained in all cases, and are identical to those seen in the earlier tetrameric HIV-1 (Figure 1D) and dimeric HIV-2 IN_NTD+CCD_ structures, although in the latter case the NTD-CCD interfaces were intramolecular [15],[30].

**Figure 2:**
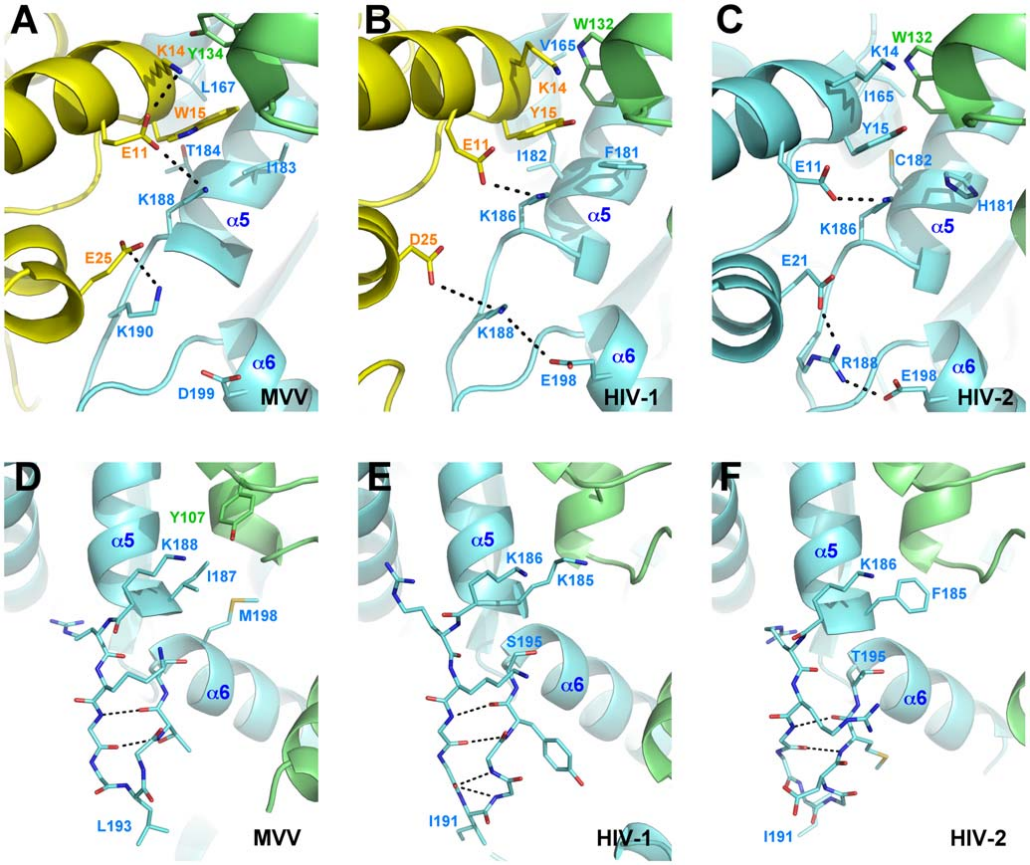
The NTD-CCD interfaces and CCD finger structures of MVV, HIV-1 and HIV-2 INs. (A–C) The NTD-CCD interface as observed in MVV IN_NTD+CCD_:LEDGF_IBD_ CF2, HIV-1 IN_NTD+CCD_ (PDB ID 1k6y) and HIV-2 IN_NTD+CCD_:LEDGF_IBD_ (PDB ID 3f9k) structures. A cartoon representation is shown, viewed from the opposite side of the tetramer to Figure 1C, with carbon atoms colored by chain as in Figure 1C and other atoms colored blue for nitrogen, red for oxygen and yellow for sulfur. Note the interface involving HIV-2 IN is intramolecular in contrast to that in the domain-swapped tetrameric MVV and HIV-1 IN structures. (D–F) Configurations of the CCD fingers in structures from panels A–C. Side and main chains of the finger residues are shown as sticks. The color scheme as in panels A–C. Hydrogen bonds are indicated with dashes. Residues discussed in the text are indicated. Note that Lys185 in the HIV-1 structure in panel E replaces Phe, naturally occurring at this position.

The NTD-CCD interfaces, observed in the structures of divergent INs, share conserved features including a well-defined salt bridge between Glu11 and Lys188 (Lys186 in HIV-1 IN; refer to Figure S1 for an MVV/HIV-1 IN sequence alignment) and hydrophobic interactions involving Trp15 (Tyr15 in HIV-1 IN) and chain A Tyr134 as well as chain B Leu167, Ile183, Thr184 and Lys188 (Trp132, Val165, Phe181, Ile182 and Lys186, respectively, in HIV-1 IN) (Figure 2A and 2B). An additional salt bridge is formed between Glu25 and Lys190, and this is reproduced in the HIV-1 IN interface as Asp25:Lys188. HIV-2 IN encodes Lys at position 25, so it cannot form the same salt bridge; instead the related Arg188 forms a salt bridge with Glu21 (Figure 2C). The conservation of the NTD-CCD interface and the resulting tetramers in crystal structures of divergent lentiviral INs strongly argues for their functional relevance.

### Closure of the flexible tetramerization interface

**Figure 3:**
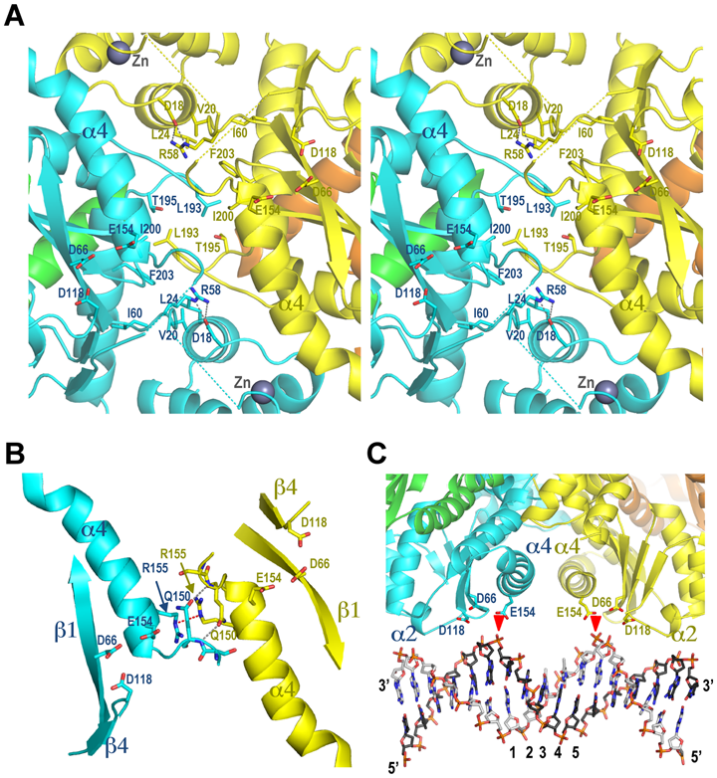
Details of the IN tetramer consistent with concerted integration. (A) Stereo view on the dimer-dimer interface in CF2, as viewed from top of the orientation in Figure 1C. The contribution of Leu193, Phe203, Ile200, Thr195, Leu24, Val20, and Ile60 residues from the inner monomers to the solvent exposed surface in CF2 structure is ∼95 Å^2^, compared to ∼280 Å^2^ in the open CF1/ABEF tetramer. Relevant side chains are shown as sticks and indicated. Gray spheres are Zn atoms. Salt bridges involving Arg58 and D18 are indicated with gray dashes. The coloring scheme is as in Figures 1 and 2. (B) Contacts involving the N-termini of inner monomeric CCD α4 helices. The structure is slightly tilted, compared to the orientation shown in panel (A). Hydrogen bonds between chain B and C Gln150 residues are shown as gray dashes. Repulsive interaction between guanidinium groups of Arg155 residues is highlighted with red dashes. (C) A conceptual model for the engagement of target DNA by a closed IN tetramer. A 17-bp DNA duplex was aligned with the pair of active sites from the inner monomers of the CF2 tetramer. The scissile phosphodiester bonds are indicated with red triangles, and the separating base pairs are numbered. Secondary structure elements discussed in the text are indicated.

Although each IN tetramer is stabilized by identical intermolecular NTD-CCD interactions, there is remarkable variation in the relative positions and orientations of the interacting dimers (Figure 1, Figure S2, Videos S1 and S2). The plasticity of the dimer-dimer interface is sufficient to allow a pair of active sites from the opposing CCD dimers in CF2 to approach 14.9 Å separation (measured as the distance between Cγ atoms of the active site Glu residues). For a comparison, the separation between the structurally-equivalent active sites in CF1/ABEF is 27.5 Å, while that in the HIV-1 IN_NTD+CCD_ structure [15] is ∼29 Å (Figure 1). In addition to the stable intermolecular NTD-CCD interactions, the tetramerization interface involves a loop connecting CCD helices α5 and α6 (residues 188–196 and 186–195 in MVV and HIV-1 respectively, Figure S1), termed finger [2]. Although rich in Gly residues, the loop adopts a constrained conformation stabilized by a network of hydrogen bonds, the aforementioned salt bridges with the NTD, and wields a hydrophobic residue at the tip (Leu193 in MVV; Ile191 in HIV) (Figure 2D–2F). Examination of the dimer-dimer interfaces within individual tetramers reveals profound differences in relative orientations and contacts made by the fingers of opposing CCD dimers (Figure 1). Notably, the fingers switch positions between CF1/CDEF and CF2 structures, with CF1/ABEF representing an intermediate state (Videos S1 and S2). The most defined, symmetric and potentially relevant interactions involving this loop are observed in the CF2 structure, where side chains of Leu193 residues nucleate a hydrophobic core, engaging Ile200, Phe203 and Thr195 from the finger of the opposing CCD dimer (Figure 3A). The chain of hydrophobic contacts propagates to involve Leu24 and Val20 from the inner NTDs and Ile60 from the CCD of the same chain and is further stabilized by a well-defined salt bridge involving Arg58 and Asp18 side chains. These interactions effectively zip the two halves of the tetramer together, bringing a pair of active sites from the inner monomers into close proximity (Videos S1 and S2). A complementary interaction between the active sites involves a symmetric pair of hydrogen bonds formed by Gln150 residues of the inner monomers (Figure 3B). Interestingly, the closure of the tetrameric structure also subtly modifies the internal configuration of the congregated active sites. Repulsive dipole-dipole interactions between realigned α4 helices, exacerbated by the close stacking of Arg155 side chains (Figure 3B), result in a slight deformation of both helices, forcing Glu154 to shift towards Asp66 and Asp118 of the same active site. For example, the distance between the Cα atoms of Glu154 and Asp66 decreases from 10.4 Å in the open CF1/ABEF and CF1/CDEF conformations to 7.7 Å in CF2. The active site separation in the closed tetramer observed in CF2 is compatible with the spacing between scissile phosphodiester bonds in B-form target DNA (Figure 3C). Hence, CF2 represents an IN tetramer conformation committed for concerted integration.

### The MVV IN-LEDGF interface

**Figure 4:**
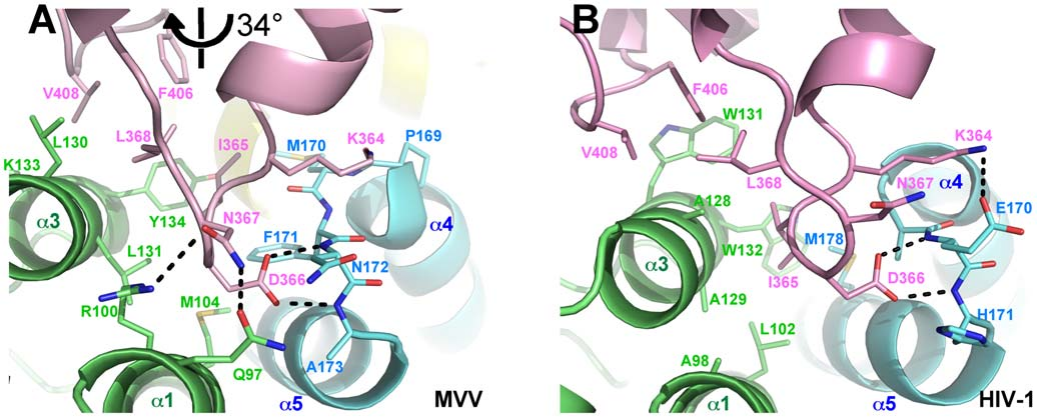
The LEDGF_IBD_:IN_CCD_ interface. Comparison of IBD:CCD interactions in MVV IN_NTD+CCD_:LEDGF_IBD_ (CF2) (A) and HIV-1 IN_CCD_:LEDGF_IBD_ (PDB ID 2b4j) (B) structures. The view is from the same side as in Figure S2D. Note the increase in inter helix spacing between MVV CCD α1 and α3, caused by the replacement of small side-chains (HIV-1 IN residues Ala98 and Ala129) with larger Arg and Leu side-chains (MVV residues 100 and 131, respectively). The resulting ∼34° rotation of the IBD is indicated by the black symbol.

Predictably, the overall architecture of the MVV IN-LEDGF interaction is similar to that described for HIV-1 and HIV-2 INs [30],[33]: it primarily involves the tip of the IBD, notably LEDGF residues Ile365 and Asp366, and a cleft at the interface of the CCD dimer. The stoichiometry of MVV IN_NTD+CCD_:LEDGF_IBD_ complexes observed in both crystal forms is 1∶1 (Figure S2), similar to that in crystals of the HIV-1 IN_CCD_:LEDGF_IBD_ complex [33]. Thus, each MVV IN CCD dimer interacts with a pair of IBDs, bound at two equivalent positions. All ten CCD:IBD interfaces observed in CF1 and CF2 structures are very similar. LEDGF Ile365 forms hydrophobic interactions with Met104, Leu131 and Tyr134 of one MVV IN chain and Met170 and Phe171 of the second IN chain (Figure 4A). These interactions are related to those observed for HIV-1, although the actual IN side-chains involved differ due to lack of sequence identity (Figure S1). As predicted [27], LEDGF Asp366 duplicates the previously described bidentate hydrogen bond with backbone amides of MVV IN residues Asn172 and Ala173 (Glu170 and His171 in HIV-1).

Lentiviral INs display surprisingly little sequence conservation at the positions directly involved in the interaction with LEDGF, itself a well-conserved protein [27],[33]. Predictably, some details of the MVV IN-LEDGF interaction show marked differences with those elaborated for HIV-1 or HIV-2 INs [30],[33] (Figure 4). One such difference occurs due to MVV encoding residues Arg100 and Leu131 in place of two Ala residues at HIV-1 IN equivalent positions 98 and 129. The bulky side-chains pry MVV IN CCD helices α1 and α3 slightly apart, enlarging the cleft occupied by the protruding IBD loop. The extra space is filled by the insertion of LEDGF side chains Asn367 and Leu368, which make hydrogen bonds with Gln97 and Arg100 and hydrophobic interactions with Leu130, Leu131 and Tyr134, respectively (Figure 4A). The result of this alternate binding orientation is a ∼34° rotation of the IBD with respect to the HIV-1 structure, centered at the site of interaction with the CCD. Consequently, Phe406 and Val408 located on the second loop of the IBD make hydrophobic interactions with MVV IN Tyr134. Such interactions would not be possible with HIV-1 IN due to an inevitable steric conflict with the side chain of Trp131; the equivalent position of MVV IN is occupied by Lys133, whose flexible side chain makes way for incoming Phe406 and Val408 (Figure 4). The rotation also allows LEDGF Lys364 to form a hydrogen bond with the carbonyl group of MVV IN Pro169 (Figure 4A). In the complex with HIV-1 IN, Lys364 forms a salt bridge with non-conserved IN residue Glu170. Additional interactions involving the positive patch on one side of the IBD structure and carboxylates of HIV-1 and HIV-2 IN NTDs are important for high affinity interaction [30]. In CF2, LEDGF residues Lys401, Lys402 and Arg405 are sufficiently close for electrostatic interactions with MVV IN Asp41, Glu10 and Glu9, respectively (not shown). However, the side chains of the interacting residues are not well defined in electron density maps.

### The dimer-dimer interface is critical for HIV-1 IN tetramerization

**Figure 5:**
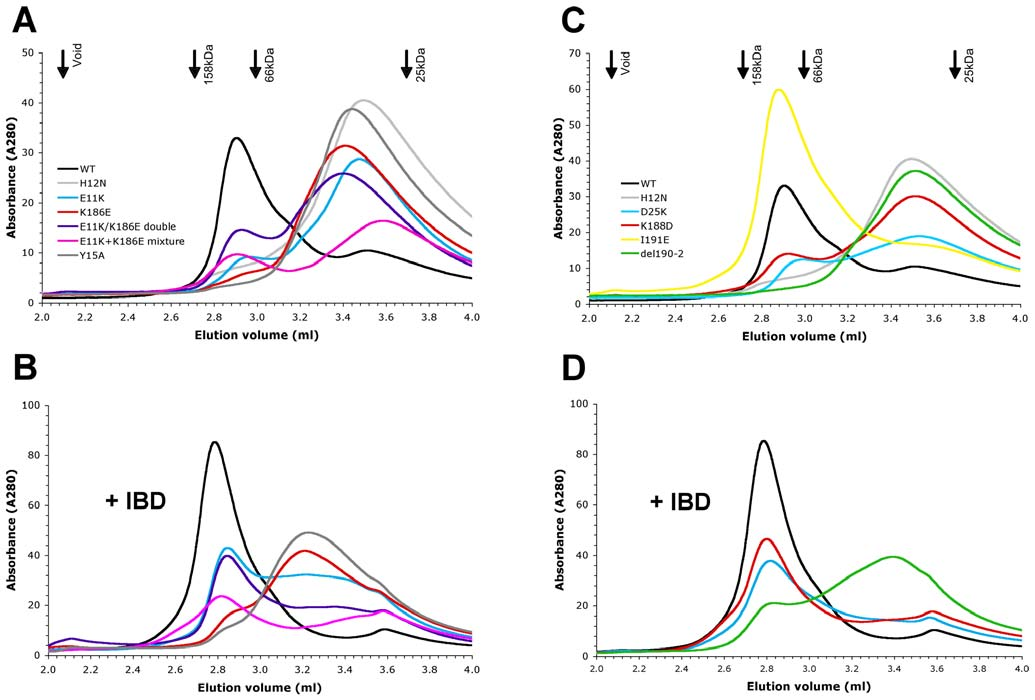
Multimerization of WT and mutant HIV-1 INs. (A) SEC elution profiles of IN proteins versus elution volumes of protein standards (black arrows). WT (black) and H12N (light gray) IN indicate the tentative volumes of tetramers and dimers, respectively. The profiles of E11K, K186E, E11K/K186E double, E11K+K186E mixture and Y15A mutants are shown in cyan, red, purple, pink and dark gray, respectively. (B) The elution profiles of the same mutant INs as in panel A, but pre-mixed with LEDGF_IBD_ prior to chromatography; colors are as in panel A. (C) SEC elution profiles of D25K, K188D, I191E and Δ190-2 mutant INs (respectively cyan, red, yellow and green) compared to the profile of WT (black) and H12N (gray) proteins. (D) Elution profiles of indicated panel C IN proteins in complex with LEDGF_IBD_.

To test the relevance of the tetramerization interface observed in the crystal structures, we designed a series of HIV-1 IN mutants. The changes were introduced at the positions predicted to be important for tetramerization by the earlier HIV-1 IN_NTD+CCD_ [15] and current MVV structures. Multimerization properties of purified proteins were studied using analytical size exclusion chromatography (SEC) (Figure 5). All proteins displayed non-ideal behavior, such as temperature-dependent interaction with Superdex and silica matrices (data not shown), and generated complex elution profiles, indicative of multiple multimeric forms. Nonetheless, in agreement with previous results [34], the elution profile of WT HIV-1 IN was consistent with a predominantly tetrameric species (Figure 5A). Preincubation of IN with an excess of LEDGF_IBD_ prior to injection resulted in a slightly earlier elution of the major species (Figure 5B). The peak shift of ∼0.15 ml was consistent with binding of four 10-kDa LEDGF_IBD_ molecules per IN tetramer. Zinc binding is essential for folding of the NTD and promotes HIV-1 IN self-association [6], [35]–[37]. Concordantly, disruption of zinc coordination by the NTD H12N mutation grossly affected the SEC elution profile (Figure 5A). Under these experimental conditions, H12N IN behaved as a dimer or a dimer-monomer mixture.

Remarkably, several mutations at the NTD-CCD interface affected HIV-1 IN self-association properties to a similar extent as the NTD-destabilizing H12N mutation. Thus, mutating Tyr15, a residue involved in several hydrophobic interactions with the CCD (Figure 2B), abolished multimerization (Figure 5A). Similarly, disrupting the Glu11:Lys186 salt bridge with single point mutations E11K or K186E resulted in pronounced shifts to lower molecular weight species (Figure 5A). Interestingly, less dramatic shifts were observed for D25K and K188D, suggesting lower importance of the Asp25:Lys188 interaction for multimerization. These results agree with an earlier report showing that the K186A change had a greater effect on tetramerization than did K188A [34] and are consistent with the crystal structures. Thus, in HIV-1 IN [15], the ε-amino group of Lys188 is shared between the carboxylates of Asp25 and Glu198, separated from either by ∼4.6 Å (Figure 2B). In contrast, the ε-amino group of HIV-1 Lys186 is only ∼3.2 Å from the carboxylate of Glu11, indicating strong bonding. In MVV IN, the Glu25:Lys190 salt bridge appears to be the stronger of the two, with the Glu11:Lys188 interaction weakened by interactions between Glu11 and Lys14 (Figure 2A). Remarkably, combining the E11K and K186E mutations in one protein led to a significant recovery of the higher-multimeric HIV-1 species, as did mixing equimolar quantities of single mutants (Figure 5A). Cross-linking with the homobifunctional reagent BS^3^ confirmed that WT HIV-1 IN existed as a predominantly tetrameric species, and that tetramerization was highly sensitive to the E11K or K186E mutation (Figure S3). Further corroborating results of the SEC experiments, partial recovery of tetramer formation was observed in equimolar mixtures of E11K and K186E mutants (Figure S3). These results demonstrate that (*i*) the contact between Glu11 and Lys186 is essential for the stability of higher-order HIV-1 IN multimers *in vitro* and (*ii*) the salt bridge between these residues can be formed intermolecularly, corroborating the NTD-CCD connectivity observed in the MVV structures.

Deletion of residues ^190^Gly-Ile-Gly^192^ from the CCD finger abrogated multimerization (Δ190-2, Figure 5C), although the I191E point mutant multimerized as well as WT (Figure 5C). Therefore, while the whole of the constrained loop structure is clearly essential for multimerization, the conserved aliphatic residue at its tip is not. LEDGF was shown to enhance HIV-1 IN tetramerization [34], an effect likely dependent on the IBD-NTD interface [30],[34]. Accordingly, preincubation with LEDGF_IBD_ led to at least partial rescue of multimerization for all NTD-CCD interface mutants (Figure 5B and 5D). These results are wholly consistent with the crystal structures (Figure S2), where LEDGF binding is expected to stabilize IN tetramers.

### The NTD-CCD interface is vital for IN enzyme activity and HIV-1 infection

**Figure 6:**
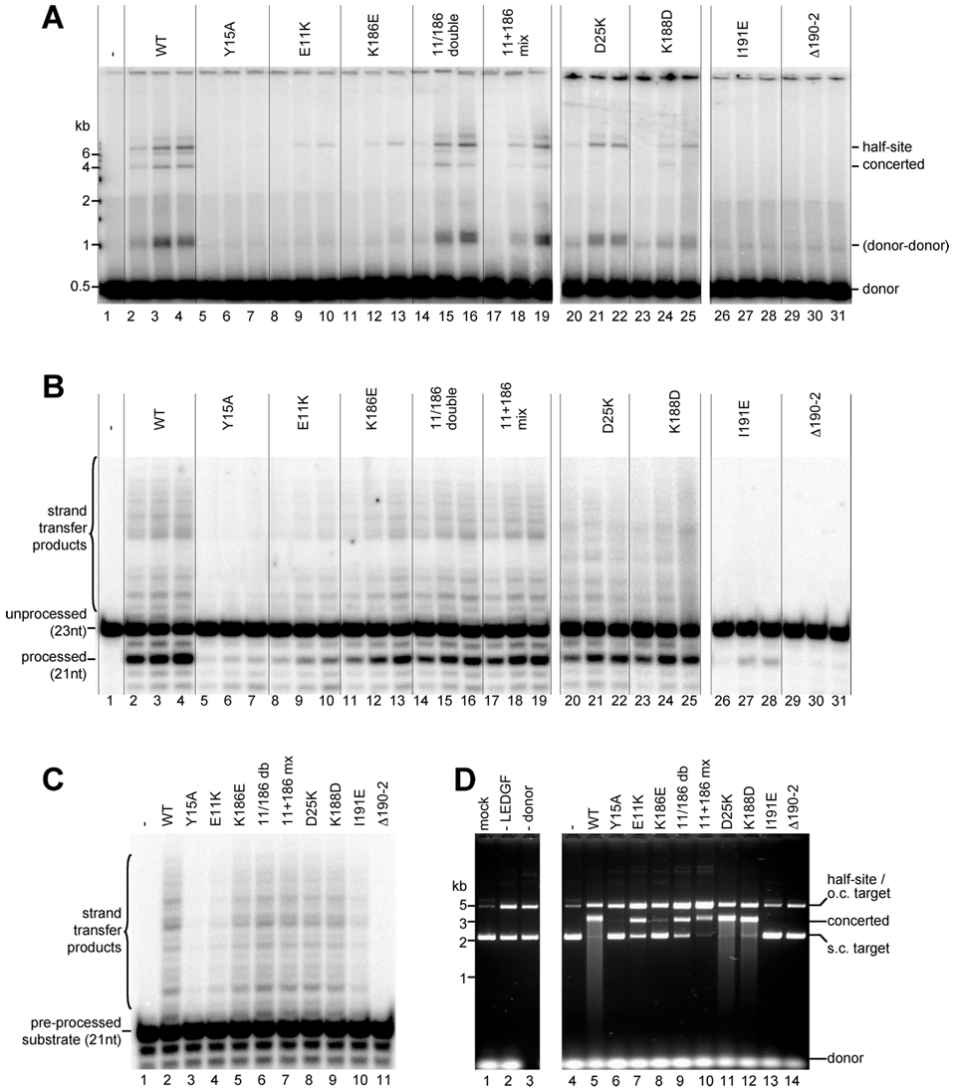
Enzymatic activities of WT and mutant HIV-1 INs. (A) Concerted integration activity. Three IN concentrations, 0.05, 0.1 and 0.2 µM (left to right) were used. The migration positions of DNA standards, the donor and the reaction products are indicated. Concerted integration of two 0.5-kb donor DNAs into the circular ∼3 kb plasmid target results in a linear ∼4 kb product, whereas half-site integration results in a tailed open circular molecule. The faint band on the gel above the first half site band is likely two half-site integration events into the same target plasmid. The fuzzy band migrating at ∼1 kb is the result of half-site integration of a donor molecule into a second donor. (B) 3′-processing and overall strand transfer activities for each IN mutant, at three different IN concentrations: 0.1, 0.2 and 0.4 µM. Migration of the radiolabeled reactive strand of the oligonucleotide substrate (23 nt), its processed form (21 nt) and the ladder of the strand transfer products are indicated. (C) Assays conducted in the same conditions as those in (B) but using pre-processed substrate, which allows the enzyme to by-pass 3′-processing. IN was used at 0.2 µM throughout. (D) LEDGF-dependent concerted integration assay using short, unprocessed (32 bp) oligonucleotides as donor DNA. Lanes 1–3 contained a mock (no protein added) reaction, LEDGF- and donor substrate-omit controls. Concerted integration in this assay results in a product migrating close to the linearized form of the target DNA, whereas half-site integration a branched form of target DNA, migrating as an open circular [30]. The smear below the concerted integration product for highly reactive INs is a result of re-targeting of the main product by additional concerted integration events. Migration of the donor DNA, supercoiled (s.c.) and open circular (o.c.) form of target DNA, and reaction products are indicated.

Next, we tested the HIV-1 IN mutants for the ability to catalyze 3′-processing and DNA strand transfer using either a blunt-ended 500-bp (Figure 6A), or blunt or pre-processed 23-bp mimic of the viral U5 DNA end (Figure 6B and 6C). The assay with the longer viral DNA substrate distinguishes concerted strand transfer reaction products from those that result from the integration of a single donor DNA end into only one strand of target DNA, whereas the oligonucleotide-based assays do not. The Y15A and Δ190-2 mutants were almost devoid of 3′-processing activity (Figure 6B), and did not produce strand transfer products in either assay format (Figures 6A–6C). Interestingly, I191E IN, which multimerized as well as WT, was attenuated for both 3′-processing (Figure 6B) and strand transfer (Figures 6A and 6C), suggesting that I191E tetramers might exist in a defective conformation. Mutants D25K and K188D functioned relatively well in 3′-processing (Figure 6B) and retained near WT strand transfer activity in the oligonucleotide assay (Figure 6C). However D25K and, to a lesser degree, K188D, displayed a specific concerted integration defect, with D25K generating half-site products at near WT level (Figure 6A).

Mutations E11K and K186E, targeting the Glu11:Lys186 salt bridge, decreased 3′-processing and strand transfer activities (Figures 6B and 6C) while completely eliminating concerted integration (Figure 6A, lanes 8–13). The importance of the salt bridge was further illustrated by the recovery of concerted integration activity to almost WT levels with the double E11K/K186E mutant (Figure 6A, lanes 14–16). This result also confirmed that the mutations do not affect the intrinsic catalytic properties of the enzyme, or its functional association with donor or target DNA. Likewise, mixing the two individual mutants (E11K+K186E), each incapable of forming intramolecular NTD-CCD interactions, recuperated concerted integration (lanes 17–19). Consistent with the observation that LEDGF binding aids IN multimerization (Figures 5B and 5D, see also [34]), the concerted integration activities of E11K, D25K, K188D, and, to a lesser extent, K186E, were rescued in the presence of the host factor (Figure 6D).

IN mutations were next introduced into the single round HIV-Luc vector, and infectivity was assessed 2 days post-infection. Based on the results with purified enzymes, E11K, K186E, and E11K/K186E mutants were initially compared to D64N/D116N (N/N) active site mutant virus. N/N supported 0.25±0.06% (*n* = 6) residual HIV-Luc infectivity, whereas E11K, K186E, and E11K/K186E faired less well, each scoring near the assay detection limit (<0.025% of HIV-Luc). This suggested that E11K, K186E, and E11K/K186E might exert class II mutant behavior: certain mutants, like N/N, are referred to as class I because they are specifically blocked at integration and accordingly support residual levels of gene expression from unintegrated DNA, whereas the majority of mutant viruses, class II, display additional reverse transcription and/or virus assembly defects [38]. The preliminary assignment of class II mutant behavior is consistent with the previously reported K186Q reverse transcription defect [39],[40].

**Figure 7:**
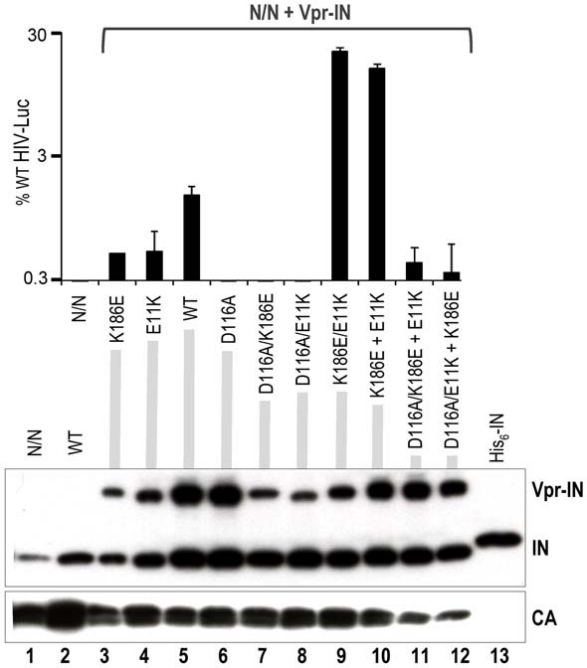
WT and mutant virus infectivity. The level of N/N active site mutant virus infection, either without added Vpr-IN (left) or with the indicated Vpr-IN protein(s), as percentage of WT HIV-Luc infectivity. Error bars indicate the variation attained from duplicate experiments (four independent infections). The western blot below the graph shows total levels of IN, uncleaved Vpr-IN and viral capsid (CA) in pelleted N/N-Luc (lane 1), HIV-Luc (lane 2) or N/N-Luc containing the indicated Vpr-INs (lanes 3–12). Lane 13 contained 3 ng recombinant His_6_-tagged IN.

The activities of class II mutant viral enzymes can be analyzed during infection via trans-incorporation of Vpr-IN fusion proteins into assembling virus particles [40],[41]. Various mutant proteins were therefore compared to Vpr-IN_WT_ for their ability to stimulate N/N-Luc infectivity. Vpr-IN_WT_ enhanced N/N-Luc infection approximately 6- to 16-fold, yielding overall infectivities that ranged from 1.4% (Figure 7) to 6.8% (data not shown) of HIV-Luc. Vpr-IN_E11K_ and Vpr-IN_K186E_ displayed partial activities, yielding 39±5.8% and 33±1.6% of Vpr-IN_WT_ function in repeat (*n* = 5) experiments (Figure 7 and data not shown). Akin to the result with purified enzymes, the Vpr-IN_E11K/K186E_ double mutant was significantly more active than either single mutant, actually outshining Vpr-IN_WT_ to restore 21.5% of HIV-Luc activity (Figure 7). Trans incorporation of separate Vpr-IN_E11K_ and Vpr-IN_K186E_ single mutants also significantly stimulated N/N-Luc, yielding 15.7% of overall HIV-Luc infectivity. Importantly, incorporating the D116A active site mutation into either Vpr-IN_E11K_ or Vpr-IN_K186E_ counteracted the stimulatory affect of the mixture (Figure 7). Immunoblotting revealed similar levels of functional and non-functional Vpr-IN protein incorporation into virions (Figure 7).

## Discussion

Retroviral INs function as multimers [16]–[19], [41]–[43]. Due to obvious structural restraints, such as distances between active sites in their dimeric CCDs, minimally a tetramer of IN would be required to carry out concerted integration of both viral DNA ends. Because a structure of a full-length IN has remained elusive, much effort is being expended to model a full-length IN tetramer based on the available partial crystal structures [15], [44]–[46]. In this work we present two crystal structures containing a two-domain construct of a divergent lentiviral IN in complex with the isolated IBD of its natural host cofactor LEDGF. Together with earlier results [15],[30], these structures elucidate the mechanism for IN tetramerization, indicate the dramatic flexibility of the IN tetramerization interface (Videos S1 and S2) and for the first time reveal a tetramer conformation that is compatible with concerted integration (Figure 3).

It is important to note that the CTD, which is also involved in IN multimerization [7],[47], is not present in our structures. Nonetheless, we were able to validate the tetramerization interface observed in the crystals using a range of functional assays with mutants of full-length HIV-1 IN. Herein we demonstrated that the main proponent of IN tetramerization is the conserved NTD-CCD interface brought about by swapping a pair of NTDs between participating IN dimers. We recently showed that within an IN dimer, the NTDs fold back onto their own CCDs [30]. In contrast, in the context of a tetramer, interacting IN dimers swap a pair of NTDs (Figure 1). Although similar connectivity was postulated earlier [15], hitherto direct evidence for NTD swapping was not available. The absence of structured NTD-CCD linkers and the open conformation of the HIV-1 IN_NTD+CCD_ tetramer described by Wang et al. [15] allow various alternative NTD-CCD connectivities (for more discussion see [30] and [2]). Detailed analyses of the NTD-CCD interfaces in the current MVV as well as earlier HIV-1 and HIV-2 IN structures [15],[30] revealed a network of conserved interactions (Figure 2) that are essential for multimerization (Figure 5). The key interaction involves a conserved salt bridge, which in HIV-1 IN is mediated by Glu11 and Lys186, and the latter residue has been shown to be important for HIV-1 IN multimerization [34],[48]. Herein we demonstrate that the Glu11:Lys186 salt bridge is functionally reversible, allowing us to significantly extend prior observations. Thus, while individual mutations of both residues abrogated tetramerization and concerted integration, mixing HIV-1 IN E11K and K186E single mutants partially recovered tetramerization (Figure 5 and S3), rescued concerted integration in vitro (Figure 6), and moreover robustly stimulated N/N-Luc infection (Figure 7). These results imply that the intermolecular NTD-CCD interface is functional. The behavior of the E11K+K186E mixture in the virus complementation assay highlights this functionality. A significant fraction of inner monomers from the N/N+Vpr-IN_WT_ mixture will contain inactivating D64N/D116N mutations, poisoning tetramer function. In the N/N+Vpr-IN_E11K_+Vpr-IN_K186E_ case, N/N IN would only be allowed to assume the role of the outer monomers to accommodate the reversible salt bridge between inner IN_E11K_+IN_K186E_ pairs. Hence the activity of the Vpr-IN_E11K_+Vpr-IN_K186E_ mixture outshines that of Vpr-IN_WT_ in this assay (Figure 7). Furthermore, because the double E11K/K186E mutant is functional, we can conclude that the mutations do not affect the intrinsic catalytic properties of the enzyme or its interactions with DNA. Not only did the double mutant E11K/K186E recover concerted integration activity and HIV-1 infection, it also supported greater levels of 3′-processing and half-site integration activities over the individual mutant proteins. This indicates that while it could be possible for a dimer of IN to catalyze 3′-processing and half-site integration, both reactions are more efficiently catalyzed by a tetramer (or possibly a larger aggregate of IN dimers). A similar conclusion was made based on kinetic studies utilizing a mutant of an alpharetroviral IN that was unable to form tetramers [49]. Furthermore, this finding is in agreement with Li and Craigie [50], who observed that 3′-processing and concerted HIV-1 integration are functionally coupled. We speculate that tetramerization could play a role in the correct organization of the active site. Indeed, closure of the tetramerization interface leads to a slight compression of the MVV IN active site, with active site residue Glu154 relocating closer to its Asp66 and Asp118 mates. In addition, IN tetramerization and engagement of the viral DNA termini are likely to be co-dependent.

Intriguing questions remain as to the nature of the class II phenotype of HIV-1 IN mutants [38]. Although E11K/K186E HIV-1 IN was fully competent to carry out concerted integration starting with blunt ended substrate (Figure 6), the virus carrying these mutations was not infectious. It is possible that Glu11 and/or Lys186 impact important noncatalytic IN function(s) at a step prior to integration, such as reverse transcription [51]. Alternatively, the mutations might disrupt interaction with a host factor that would engage the outer IN monomers of the tetramer during integration. It is important to note that the IN tetramer structure contains two structurally and functionally-distinct pairs of IN subunits, with the inner pair (painted cyan and yellow in Figure 1) swapping their NTDs and providing the active sites, and the other pair (green and orange) playing a supporting role. Therefore, many residues in the IN sequence likely have two distinct functions.

The current MVV and the earlier HIV-1 IN [15] structures (Figure 1), as well as our analyses of the Δ190-2 mutant, clearly indicate that the CCD finger is involved in multimerization. Similarly, alterations within the CCD finger structure impaired tetramerization of alpharetroviral IN [48]. Truncation of the constrained loop structure is expected to affect salt bridges involving HIV-1 Lys186 and Lys188 side chains, and thus the crucial intermolecular NTD-CCD interface. The significance of the aliphatic residue at the tip of the finger structure (Ile191 in HIV-1 or Leu193 in MVV) is highlighted by its conservation in all lentiviruses. A substitution of HIV-1 IN Ile191 for Glu produced a protein that was able to multimerize (Figure 5), but was essentially devoid of enzymatic activity (Figure 6). These results are consistent with the importance of the aliphatic residue for the formation of the closed tetramer conformation, represented by the CF2 structure, where a pair of Leu193 residues from opposing CCD fingers nucleate a hydrophobic core at the dimer-dimer interface (Figures 1 and 3A).

Superposing partial HIV-1 IN structures onto the CF2 MVV structure results in a plausible full-length tetrameric model devoid of significant steric conflicts (Figure S4). Although the majority of the residues involved in the closure of the dimer-dimer interface are not conserved between MVV and HIV-1 INs (Figure S1), the model suggests a potential role of HIV-1 IN residue Tyr194 in formation of the closed structure via hydrophobic interactions with Ile191 from the opposing dimer. The conformational variability of the dimer-dimer interface described here suggests that the committed IN tetramer is likely stabilized via IN-DNA interactions. It is noteworthy that the synaptic Tn5 transposase:DNA complex is primarily stabilized via protein-DNA interactions [20].

An earlier model based on the open conformation of HIV-1 IN tetramer suggested that target DNA would bind into the cleft between widely separated active sites [15],[44]. This implies that the active sites would approach target DNA duplex from opposing sides, a configuration not easy to reconcile with the size of target DNA duplications flanking integrated proviruses. On the other hand, the closed tetramer conformation would preclude target DNA access to the interior of the dimer-dimer interface. We speculate that the target duplex binds roughly along the vector connecting the active sites, affording them direct access to the scissile phosphodiester bonds located across the major groove (Figures 3C and S4). This binding mode is supported by findings of Katzman and colleagues, who demonstrated that HIV-1 IN residue Ser119, located within CCD α2, is involved in target DNA capture [52],[53]. More recent results from this laboratory further confirm a target DNA binding platform extending along this direction [54]. The locations of the CTDs in the current model (Figure S4) are compatible with a role in binding viral DNA termini. It is noteworthy that although the CCD-CTD linker adopted alpha helical conformation in the structure of the HIV-1 IN_CCD+CTD_ fragment [13], similar studies with INs from Rous sarcoma and simian immunodeficiency viruses [55],[56] highlighted significant flexibility of this region. DNA binding moreover induced considerable structural rearrangements within the CCD-CTD linker of HIV-1 IN [57]. Hence positions and orientations of the CTDs within the tetramer cannot be directly inferred from the available partial structures.

Because the current MVV (Figure S2) and earlier HIV-1 IN [15] tetrameric structures disagree on the locations of the outer NTDs, their roles remain uncertain. In particular, the NTD-NTD interfaces observed in MVV CF1 tetramers (Figure S2) differ both from each other and from those observed in HIV-1 IN_NTD+CCD_ or the isolated HIV-1 NTD dimer in solution [12]. These interfaces likely represent packing artifacts in crystal structures, which contain continuous chains of dimers linked by tetramerization interfaces, with the outer NTDs in one tetramer assuming roles of inner NTDs in another (not shown). In contrast, the tetramer in CF2 is isolated and does not have NTD:NTD contacts, with the outer NTDs folding back to lock onto the connected CCDs (Figure S2D). We expect that the outer NTDs would reveal their role in a tetramer of full-length retroviral IN or within its complex with DNA.

## Materials and Methods

### Recombinant DNA and proteins

The plasmid pCDF-MVV-IN_NTD+CCD_, used for bacterial expression of non-tagged MVV IN_NTD+CCD_, was made by ligating a PCR fragment encoding residues 1–219 of IN from molecular clone KV1772 [58] between NcoI and XhoI sites of pCDF-Duet1 (Novagen). The MVV IN_NTD+CCD_:LEDGF_IBD_ complex, used for crystallography, was produced and purified essentially as described previously for HIV-2 IN_NTD+CCD_:LEDGF_IBD_ [30]. Briefly, MVV IN_NTD+CCD_ was co-expressed with His_6_-SUMO-tagged LEDGF_IBD_ in *Escherichia coli* PC2 cells [27] transformed with pCDF-MVV-IN_NTD+CCD_ and pES-IBD-3C7 [30]. The protein complex, enriched by absorption to NiNTA agarose (Qiagen), was treated with SUMO and human rhinovirus (HRV) 14 3C proteases to release LEDGF_IBD_ from the N-terminal His_6_-SUMO tag and the C-terminal flexible tail, respectively. The complex, purified by SEC on a Superdex-200 column in 1 M NaCl, 50 mM Tris HCl, pH 7.4, was supplemented with 5 mM DTT, concentrated to 12–15 mg/ml and stored on ice.

For purification of isolated LEDGF_IBD_, *E. coli* PC2 cells transformed with pES-IBD-3C7 [30] and grown in LB medium to an A_600_ of 0.8–1.0 were induced with 0.25 mM isopropyl-thio-β-D-galactopyranoside at room temperature for 3–4 h. Bacteria were lysed by sonication in 500 mM NaCl, 0.5 mM PMSF, 20 mM imidazole, 50 mM Tris HCl, pH 7.4, and the pre-cleared lysate was incubated with NiNTA agarose (Qiagen). The resin was extensively washed with 20 mM imidazole, 500 mM NaCl, 50 mM Tris HCl, pH 7.4. The protein, eluted in 200 mM imidazole, 500 mM NaCl, 50 mM Tris HCl, pH 7.4, was supplemented with 5 mM DTT and SUMO protease (20 mg protease per mg protein) [30],[59] and dialyzed overnight against cold 250 mM NaCl, 25 mM Tris HCl pH 7.4, 5 mM DTT, 40 mM imidazole. The protease and the released His_6_-SUMO tag were depleted by passing the sample through a 5-ml HisTrap column (GE Healthcare). To remove the disordered C-terminal tail (residues 436–471) [60], the protein was digested with HRV14 3C protease (20 mg protease per mg protein) at 7°C in the presence of 10 mM DTT. Minimal LEDGF_IBD_ was then purified by chromatography through a HiLoad 16/60 Superdex-200 column (GE Healthcare).

To obtain HIV-1 IN mutants, the corresponding changes were introduced into pCPH6P-HIV1-IN [30] using quick-change procedure (Stratagene). Full-length LEDGF, HIV-1 IN and the mutant proteins were produced in bacteria and purified as previously described [27],[30]. All proteins used in activity assays and analytical chromatography experiments were tag-free.

### Crystallization and structure determination

Hanging drop vapor diffusion crystallization experiments were conducted at 18°C, mixing 1 µl MVV IN_NTD+CCD_:LEDGF_IBD_ complex (5 mg/ml in 400 mM NaCl, 2 mM DTT, 20 mM Tris HCl, pH 7.4) with 1 µl of a reservoir solution. CF1 was obtained using a reservoir solution of 25–30% (w/v) Jeffamine M600 (Hampton Research) in 100 mM Bis-Tris propane-HCl, pH 6.6. The crystals, grown over 5–10 days to a size of ∼50×50×30 µm, were cryoprotected in the reservoir solution supplemented with 20% (v/v) glycerol and frozen by immersion in liquid nitrogen. CF1 belonged to space group P2_1_ with unit cell constants a = 91.1 Å, b = 148.9 Å, c = 91.1 Å, α = γ = 90°, β = 113.4°. A dataset, collected at 100 K on beamline I04 of the Diamond Light Source (Oxford, UK), was integrated and scaled in XDS [61] to 3.28 Å (Table 1). The structure was solved by molecular replacement using Molrep [62] with three search models: HIV-1 IN CCD dimer (residues 50–212, from 2b4j), followed by LEDGF IBD (residues 347–426, 2b4j), and finally HIV-1 IN NTD (residues 1–43, 1k6y). The resulting model containing six IN and six LEDGF chains was refined using rigid body, maximum likelihood and simulated annealing routines as implemented in Phenix [63] with manual building in Coot [64]. Group isotropic B factors (one per residue) and 6-fold non-crystallographic symmetry (NCS) were applied throughout; translation, libration and screw-rotation (TLS) displacements [65] were accounted for towards the end of the refinement. The final refined model has good geometry and R_work_/R_free_ of 21.3/25.5% (Table 1).

CF2 was obtained using a reservoir solution containing 0.7–0.9 M (NH_4_)_2_HPO_4_, 2.5% Jeffamine M600 and 100 mM Bis-Tris propane-HCl, pH 7.0. Crystals, cryoprotected in the reservoir solution supplemented with 20% glycerol, were frozen by immersion in liquid nitrogen, and the data were acquired at 100 K on the Diamond Light Source beamline I02. CF2 belongs to space group P2_1_ with unit cell constants a = 102.7 Å, b = 83.0 Å, c = 115.3 Å, α = γ = 90°, β = 101.8°. Diffraction intensity data were corrected for the observed lattice translocation defect [66]; full details of the detwinning procedure will be reported elsewhere (S.H., P.C., J.W., submitted for publication). The structure was solved by molecular replacement, using Molrep with the MVV IN CCD dimer (from CF1) as a search model, followed by IBD (from 2b4j) and MVV IN NTD. Two CCD dimers were found to form a tetramer with four associated NTDs and IBDs. Following additional cycles of building, TLS and restrained refinement in Refmac [67] the final model had R_work_/R_free_ of 22.6/25.5% and good geometry (Table 1). Weighted 2Fo-Fc electron density maps for chain B of CF1 (showing the ordered NTD-CCD linker) and for three parts of the CF2 structure (NTD:CCD and IBD:CCD interfaces, as well as the chain B active site with an associated phosphate ion) are shown in Figure S5. Transition states between observed conformations of the MVV IN tetramer (Videos S1 and S2) were simulated using Yale Morph Server [68]. Protein structure images and animations were generated using PyMOL software (DeLano, W.L., http://www.pymol.org). The coordinates and structure factors for CF1 and CF2 have been deposited in the Protein Data Bank with pdb IDs 3hpg and 3hph, respectively. Raw diffraction images are available upon request.

### Analytical SEC and cross-linking

SEC was carried out using a 4.3-ml KW403-4F column (Shodex) attached to an ÄKTA Purifier system (GE Healthcare). The column was immersed in ice and operated at 0.275 ml/min in 750 mM NaCl, 10 mM MgCl_2_ and 20 mM HEPES-NaOH, pH 7.0. Thirty-five µl IN (WT or mutant) diluted to 0.6 mg/ml in gel filtration buffer supplemented with 25 µM ZnCl_2_ and 2.8 mM CHAPS was injected into the column. Where indicated, 0.3 mg/ml LEDGF_IBD_ was pre-incubated with IN on ice for 5 min prior to injection.

For cross-linking, 6 µl WT, E11K or K186E IN, or an equimolar IN mutant mixture (0.54 mg/ml protein in 1 M NaCl, 5 mM DTT, 7.5 mM CHAPS, 25 mM Hepes-NaOH, pH 7.5) was diluted with 21 µl reaction buffer (0.75 M NaCl, 2 mM MgSO_4_, 25 µM ZnCl_2_, 25 mM Hepes-NaOH, pH 7.5). Cross-linking was initiated by addition of 4 µl BS^3^ (Pierce; fresh 15–1.7 mM stock in water). Where indicated, reactions were supplemented with 0.3% SDS prior to addition of the cross-linking reagent. Reactions, allowed to proceed for 30 min at 18°C, were stopped by addition of Laemmli SDS PAGE sample buffer. The products were separated in Novex 10–20% Tricine SDS PAGE gels (Invitrogen) and detected by staining with Sypro Orange (Invitrogen).

### Integrase enzymatic assays

Oligonucleotide-based 3′-processing assays were carried out as previously described [40]. Briefly, blunt 23-bp DNA substrate was obtained by annealing 5′-end labeled 5′-CAGTGTGGAAAATCTCTAGCAGT with 5′-ACTGCTAGAGATTTTCCACACTG. Reactions (20 µl) contained 0.1–0.4 µM IN, 25 nM substrate DNA in 20 mM NaCl, 7.5 mM MnCl_2_, 10% glycerol, 10 mM β-mercaptoethanol, 0.1 mg/ml BSA and 25 mM MOPS-NaOH, pH 7.2. Reactions, initiated by addition of 0.5 µl IN in 750 mM NaCl, 5 mM DTT and 10 mM Tris-HCl, pH 7.4 (DB), were allowed to proceed for 1 h and were stopped by addition of 15 mM ethylenediaminetetraacetic acid (EDTA) and 0.3% sodium dodecyl sulfate (SDS). Products, separated on denaturing 17% polyacrylamide gels, were visualized and quantified by phosphor autoradiography using a Storm 860 imager. Strand transfer reactions using pre-processed donor DNA were carried out under the same conditions, except the 5′-CAGTGTGGAAAATCTCTAGCA oligonucleotide was radiolabeled.

The concerted integration assay [50],[69] used pGEM-9Zf(-) as target and 5′- end labeled 500-bp HIV-1 RU5 fragment [30] as donor. Reactions (25 µl) contained 50–200 nM IN, 15 nM donor DNA and 11 nM pGEM in 100 mM NaCl, 10 mM MgSO_4_, 5 mM DTT, 20 µM ZnCl_2_, 5% dimethyl sulfoxide (DMSO), 12% polyethylene glycol (PEG) 6000 and 20 mM HEPES-NaOH, pH 7.5. Reactions were started with the sequential addition of donor DNA, target DNA, 1 µl IN in DB and 1.25 µl DMSO, followed by a 2–4 min pre-incubation at room temperature before addition of 6 µl 50% PEG6000. Reactions, incubated for 1 h at 37°C, were stopped by addition of 15 mM EDTA and 0.3% SDS. The products, deproteinized by digestion with proteinase K and precipitation with ethanol, were analyzed by electrophoresis through 1.5% agarose gels in Tris-acetate buffer. Products were visualized in dried gels using a Storm 860 imager (GE Healthcare).

The LEDGF-dependent concerted integration assay [30] used blunt 32-bp donor DNA substrate, obtained by annealing oligonucleotides 5′-CCTTTTAGTCAGTGTGGAAAATCTCTAGCAGT and 5′-ACTGCTAGAGA TTTTCCACACTGACTAAAAGG, and supercoiled pGEM target. Reactions (40 µl) contained 1 µM IN, 0.6 µM LEDGF, 0.6 µM donor DNA and 34 nM pGEM in 20 mM Hepes-NaOH pH 7.4, 10 mM DTT, 110 mM NaCl, 5 mM MgSO_4_ and 4 µM ZnCl_2_. Reactions were initiated by the addition of 2 µl IN in DB, followed by a 10-min incubation at room temperature, before addition of 2 µl LEDGF in DB. Reactions were allowed to proceed for 30 minutes at 37°C and stopped by addition of 25 mM EDTA and 0.5% SDS. DNAs recovered by ethanol precipitation following deproteinization with 40 µg proteinase K for 1 h at 37°C were resolved by electrophoresis through 1.5% agarose gels and detected by staining with ethidium bromide.

### HIV-1 infection

Single-round HIV-1 strain NLX.Luc.R- carrying luciferase in place of *nef* (HIV-Luc) and either WT or D64N/D116N (N/N) active site mutant IN was pseudotyped with vesicular stomatitis virus G envelope glycoprotein as described [22],[30],[40]. WT or mutant IN protein was incorporated in trans during virus assembly by co-transfecting pRL2P-Vpr-IN plasmids [40]. Resulting cell-free virus titers were determined by reverse transcriptase incorporation of [α-^32^P]TTP. HeLa-T4 cells [70] (40,000 in 12 well plates) infected in duplicate with 10^6^ RT-cpm in 0.8 ml for 8 h were washed, lysed at 44 h post-infection, and luciferase activities were normalized to total protein content. Levels of virion-associated IN and capsid proteins were compared using western blotting as described [71],[72].

### Sequence analysis of LEDGF/p75 cDNA from *Ovis aries*

GenBank entries EE831415 and EE774051, identified using translated BLAST to span portions of *Ovis aries* LEDGF/p75 cDNA, were used to design oligonucleotide primers to isolate its entire coding region. To this end, total RNA prepared from phytohemagglutinin-stimulated sheep peripheral blood mononuclear cells was reverse-transcribed using Superscript III (Invitrogen) and gene-specific primer 5′-CTATCAATTACACATTAACATACACAC. A fragment spanning the entire coding region of sheep LEDGF cDNA was PCR-amplified using EasyA DNA polymerase (Stratagene) and primers 5′-CCTGAAACATGACTCGCGACTTCAAACC, 5′-ACTTCTCAAATGTTCTTTATATTCCAGG. The sequence determined using a pool of products from four independent amplification reactions was deposited with GenBank with the accession number FJ497048 (RefSeq: NM_001143892).

## Supporting Information

Figure S1 Amino acid sequence alignment of MVV and HIV-1 INs. Invariant residues are highlighted in bold print; residues constituting the HHCC and D,D-35-E motifs are blue and red, respectively. Blue triangles indicate HIV-1 IN residues targeted by mutagenesis in this study. Residues involved in the interaction with LEDGF are highlighted in pink, those involved in the intermolecular NTD-CCD interface in cyan, and those participating in the closure of the MVV IN tetramer in pale green; note that MVV Tyr134 and HIV-1 Trp132 are both pink and cyan. NTD, CCD and CTD spans are indicated, with the CCDs boxed. Residue numbering above and below the alignment corresponds to the MVV and HIV-1 sequences, respectively. Secondary structure elements, shown atop the alignment, are numbered starting from the beginning of each domain. Note that the CTD is not present in the MVV structures. HIV-1 secondary structure was extracted from PDB entries 1k6y and 1ex4. This figure was prepared using ESPript (http://espript.ibcp.fr/).
(0.70 MB PDF)

Figure S2 Various tetrameric arrangements of MVV IN observed in CF1 (A–C) and CF2 (D). For each structure the tetrameric chains are colored as in Figure 1 of the main text and are aligned with respect to the green and cyan CCD dimer; LEDGF chains are pink. Active site residues Asp66, Asp 118 and Glu154 are indicated by red sticks. For the majority of inner monomers, NTD-CCD connectivities are indicated by dashes. The ordered NTD-CCD linker for CF1 chain B is shown as backbone stick representation in panel C.
(5.39 MB PDF)

Figure S3 Cross-linking experiments. WT (lanes 1–4), E11K (lanes 5–8), or K186E (lanes 9–12) HIV-1 IN (3 µM), or a mixture of the E11K and K186E mutants (1.5 µM each) (lanes 13–16) were incubated with 2 - 0.2 mM BS^3^, in the presence (lanes 1, 5, 9, 13) or absence of 0.3% SDS, as indicated. The reaction products, resolved in SDS PAGE gels, were detected by staining with Sypro Orange. Positions of molecular weight markers are indicated to the left of the gel image. To the right of the gel migration positions of the tetramers as well as the products of partial cross-linking (monomers, dimers, and trimers) are shown. The gel is shown in reverse contrast.
(3.96 MB PDF)

Figure S4 Composite model of a full-length HIV-1 IN tetramer in closed conformation. The model was obtained by superposition of partial HIV-1 IN_NTD+CCD_ (PDB ID 1k6y) and IN_CCD+CTD_ (PDB ID 1ex4) structures onto the IN_NTD+CCD_ tetramer observed in CF2 (Figure 1C, Figure S2D). The CCDs and inner NTDs are colored as in Figure 1, LEDGF chains are omitted for clarity. The outer NTDs belonging to the green and orange IN chains are shown pale green and pale orange, respectively. The CTD regions derived from HIV-1 IN_CCD+CTD_ are gray. Note that the CCD-CTD linker region, here shown in alpha helical conformation, is flexible (see main text for more discussion) and is likely to adopt a different conformation in the context of the full-length protein. Four orientations of the model, related by 90° rotations, are shown. The orientation on the top left is identical to that of the CF2 tetramer in Figure 1C. The lower right inset shows a magnified view of the dimer-dimer interface, with residues Ile191 and Tyr194 shown as sticks. The other inset magnifies the potential target DNA binding face, with Ser119 and Glu152 residues from the inner monomers highlighted. Red triangles mark the scissile phospodiester bonds across the major groove.
(3.43 MB PDF)

Figure S5 Examples of weighted 2Fo-Fc electron density maps for the refined structures. (A) IN chain B in CF1. Electron density, displayed as chicken wire, is colored blue for the NTD-CCD linker region (residues 44–61) and gray for the rest of the chain. The protein is shown as sticks and semitransparent cartoon. The NTD, CCD and linker are indicated. (B) The interface involving chain C NTD and the AB CCD dimer in CF2. (C) The interface of LEDGF chain E with AB CCD in CF2. (D) Active site of IN chain B with an associated phosphate ion in CF2. Note that a phosphate ion has been observed in a structurally identical position in two HIV-1 IN structures (PDB IDs 1k6y and 2b4j). The map in panel A is contoured at 1σ and those in panels B–D at 1.2σ. Carbon atoms are colored by chain as indicated in the legends to the right, and other atoms are colored blue for nitrogen, red for oxygen, yellow for sulfur, or orange for phosphorus. The gray sphere is zinc; red spheres are water molecules.
(9.87 MB PDF)

Video S1 Simulation of transitions between the open and closed conformations of the MVV IN tetramer (side view). Experimentally determined structures CF1/CDEF, CF1/ABEF and CF2 correspond to the first, middle and the last frames of the animation, respectively. IN chains are shown as cartoons; residues discussed in the main text are shown in ball-and-stick style. The color code is preserved from Figure 1 of the main text. Running numbers show separation of the active sites (measured as distance between Cγ atoms of Glu154 residues in cyan and yellow chains). Asp66, Asp118 and Glu154 in the inner monomers are collectively indicated as DDE motifs. Residues 148–151 from the inner monomers (cyan and yellow) are omitted for clarity. Note a slight deformation of α4 helices and compression of the active sites towards the end of the animation. Transitions states were interpolated using Yale Morph Server (http://molmovdb.org/), and the movie was created with PyMOL (http://pymol.sourceforge.net/).
(4.83 MB MOV)

Video S2 Simulation of transitions between the open and closed conformations of MVV IN tetramer (view from top). Same as in Video S1, with the tetramers viewed from top, as in Figure 1 of the main text.
(4.97 MB MOV)
