# Supplementary figures and images for: Structural Basis for Functional Tetramerization of Lentiviral Integrase
Source: PLoS Pathog. 2009 Jul 17;5(7):e1000515. doi: 10.1371/journal.ppat.1000515 (PMC2705190; doi:10.1371/journal.ppat.1000515)

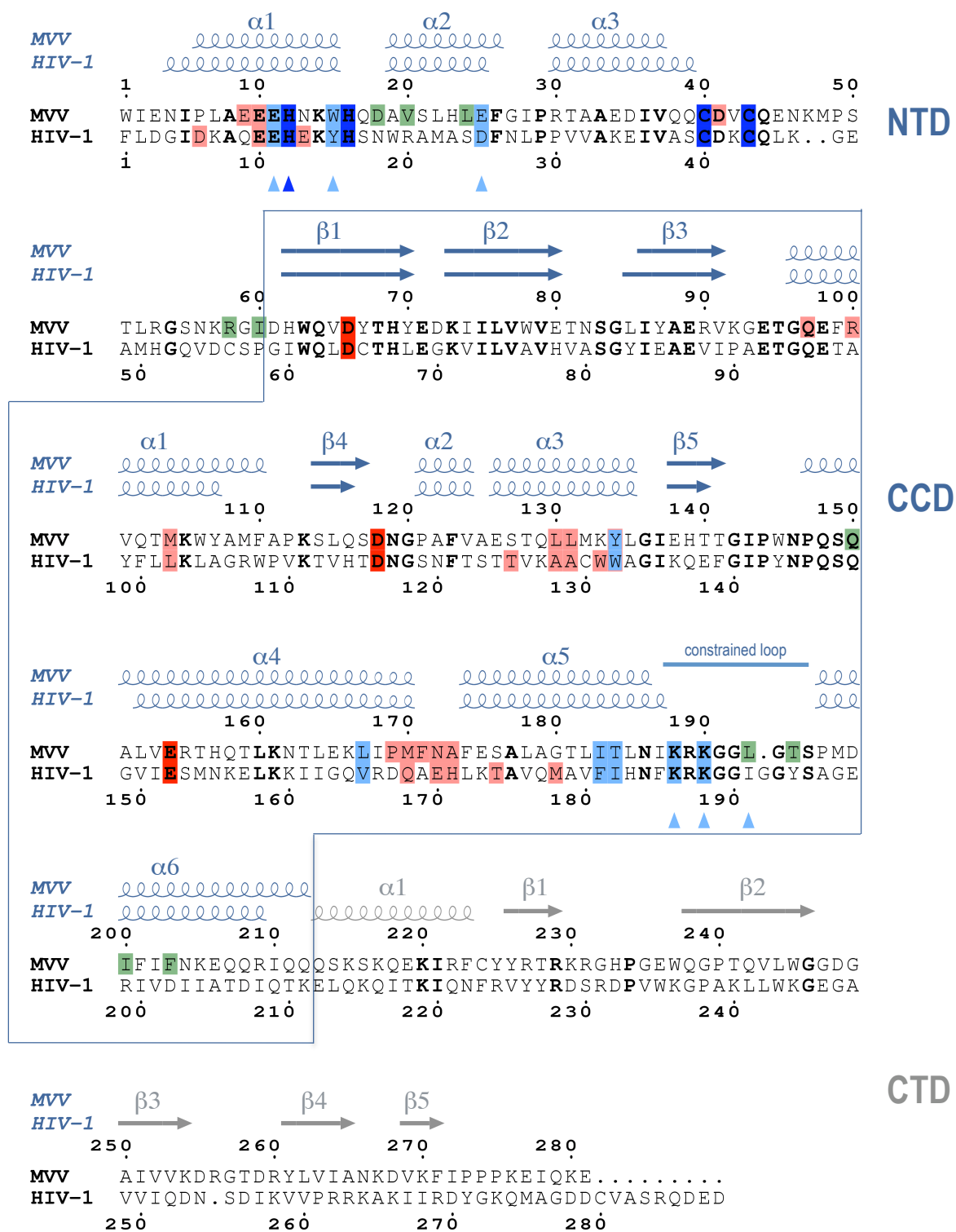

Supplement: Figure S1 — Amino acid sequence alignment of MVV and HIV-1 INs. Invariant residues are highlighted in bold print; residues constituting the HHCC and D,D-35-E motifs are blue and red, respectively. Blue triangles indicate HIV-1 IN residues targeted by mutagenesis in this study. Residues involved in the interaction with LEDGF are highlighted in pink, those involved in the intermolecular NTD-CCD interface in cyan, and those participating in the closure of the MVV IN tetramer in pale green; note that MVV Tyr134 and HIV-1 Trp132 are both pink and cyan. NTD, CCD and CTD spans are indicated, with the CCDs boxed. Residue numbering above and below the alignment corresponds to the MVV and HIV-1 sequences, respectively. Secondary structure elements, shown atop the alignment, are numbered starting from the beginning of each domain. Note that the CTD is not present in the MVV structures. HIV-1 secondary structure was extracted from PDB entries 1k6y and 1ex4. This figure was prepared using ESPript (http://espript.ibcp.fr/). (0.70 MB PDF) [file ppat.1000515.s001.pdf]

**A**

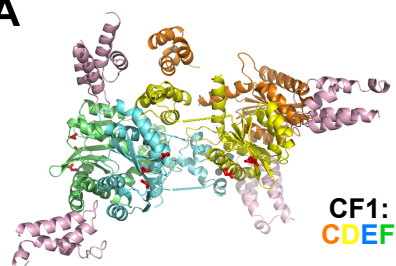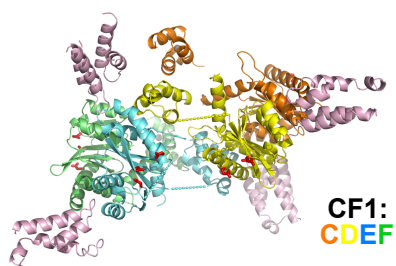

**B**

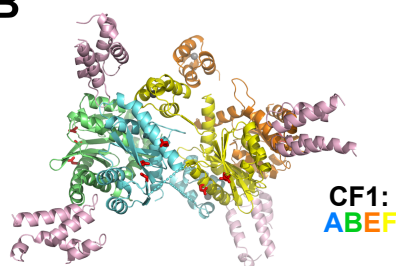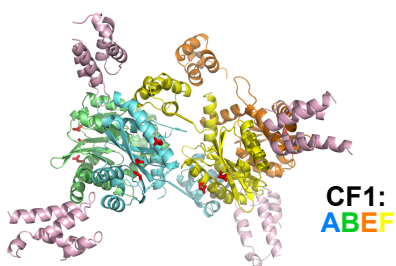

**C**

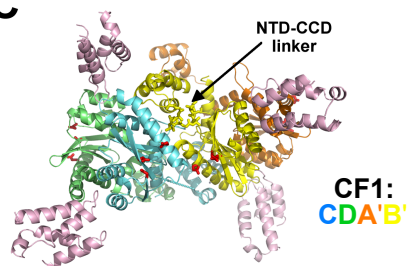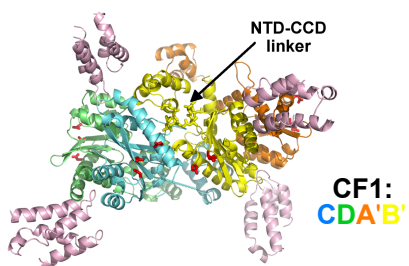

**D**

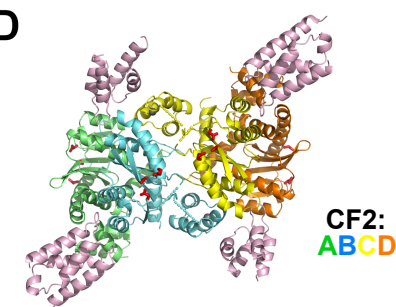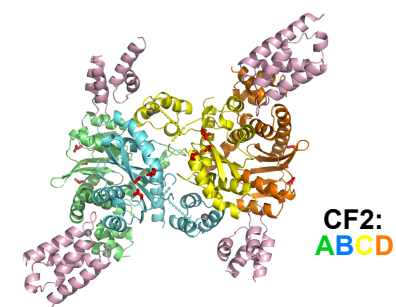

Supplement: Figure S2 — Various tetrameric arrangements of MVV IN observed in CF1 (A–C) and CF2 (D). For each structure the tetrameric chains are colored as in Figure 1 of the main text and are aligned with respect to the green and cyan CCD dimer; LEDGF chains are pink. Active site residues Asp66, Asp 118 and Glu154 are indicated by red sticks. For the majority of inner monomers, NTD-CCD connectivities are indicated by dashes. The ordered NTD-CCD linker for CF1 chain B is shown as backbone stick representation in panel C. (5.39 MB PDF) [file ppat.1000515.s002.pdf]

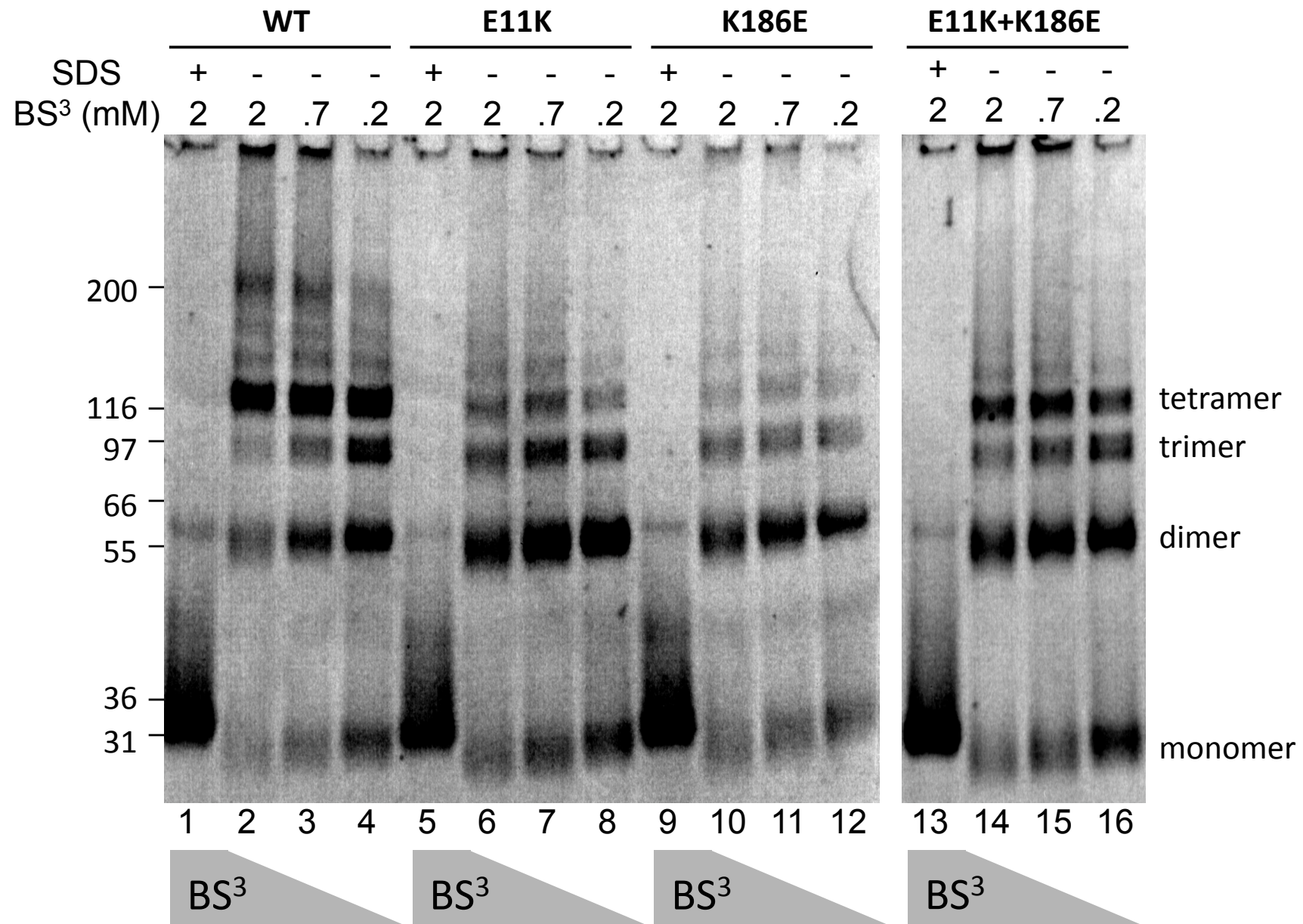

Supplement: Figure S3 — Cross-linking experiments. WT (lanes 1–4), E11K (lanes 5–8), or K186E (lanes 9–12) HIV-1 IN (3 µM), or a mixture of the E11K and K186E mutants (1.5 µM each) (lanes 13–16) were incubated with 2 - 0.2 mM BS3, in the presence (lanes 1, 5, 9, 13) or absence of 0.3% SDS, as indicated. The reaction products, resolved in SDS PAGE gels, were detected by staining with Sypro Orange. Positions of molecular weight markers are indicated to the left of the gel image. To the right of the gel migration positions of the tetramers as well as the products of partial cross-linking (monomers, dimers, and trimers) are shown. The gel is shown in reverse contrast. (3.96 MB PDF) [file ppat.1000515.s003.pdf]

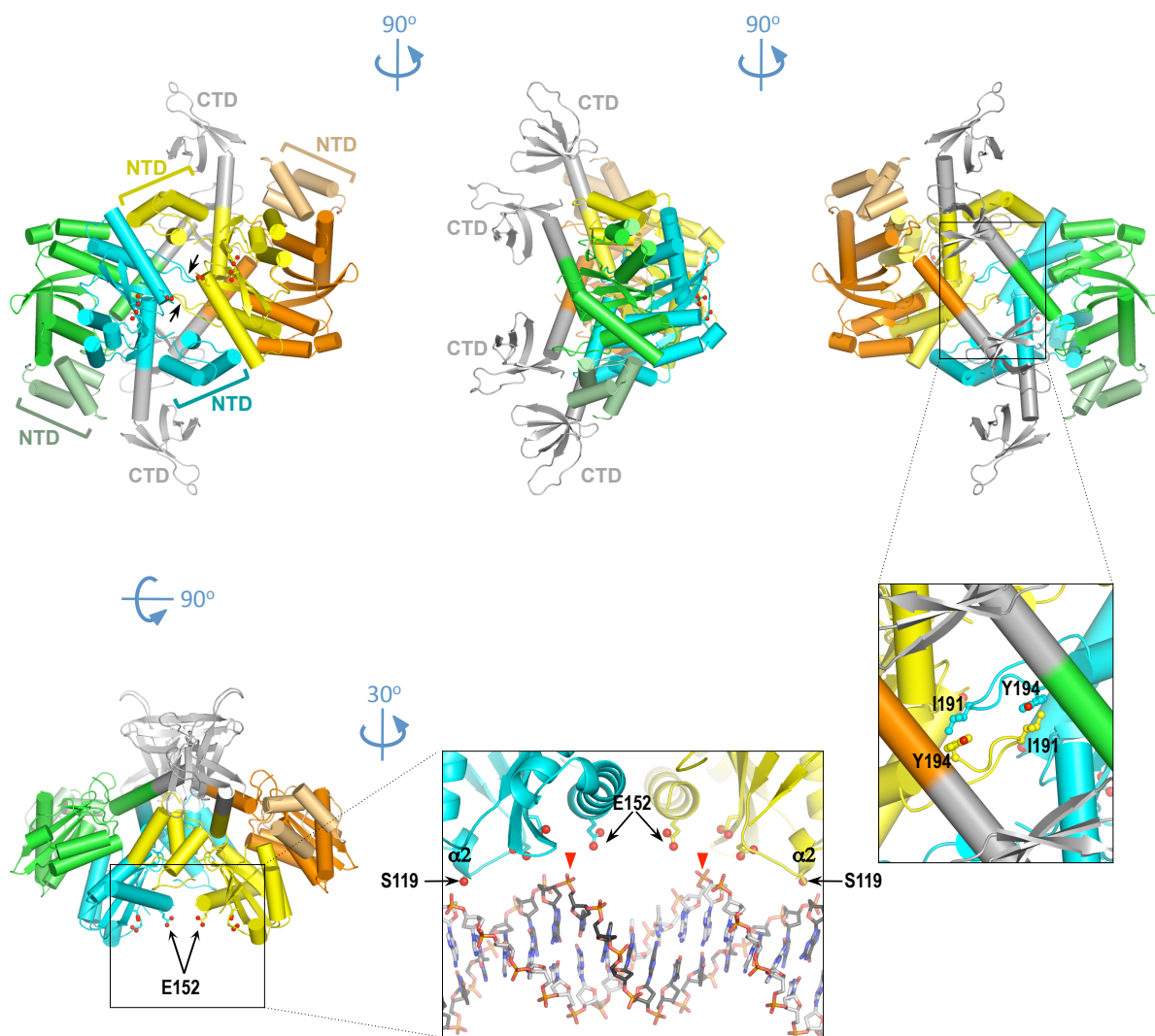

Supplement: Figure S4 — Composite model of a full-length HIV-1 IN tetramer in closed conformation. The model was obtained by superposition of partial HIV-1 INNTD+CCD (PDB ID 1k6y) and INCCD+CTD (PDB ID 1ex4) structures onto the INNTD+CCD tetramer observed in CF2 (Figure 1C, Figure S2D). The CCDs and inner NTDs are colored as in Figure 1, LEDGF chains are omitted for clarity. The outer NTDs belonging to the green and orange IN chains are shown pale green and pale orange, respectively. The CTD regions derived from HIV-1 INCCD+CTD are gray. Note that the CCD-CTD linker region, here shown in alpha helical conformation, is flexible (see main text for more discussion) and is likely to adopt a different conformation in the context of the full-length protein. Four orientations of the model, related by 90° rotations, are shown. The orientation on the top left is identical to that of the CF2 tetramer in Figure 1C. The lower right inset shows a magnified view of the dimer-dimer interface, with residues Ile191 and Tyr194 shown as sticks. The other inset magnifies the potential target DNA binding face, with Ser119 and Glu152 residues from the inner monomers highlighted. Red triangles mark the scissile phospodiester bonds across the major groove. (3.43 MB PDF) [file ppat.1000515.s004.pdf]

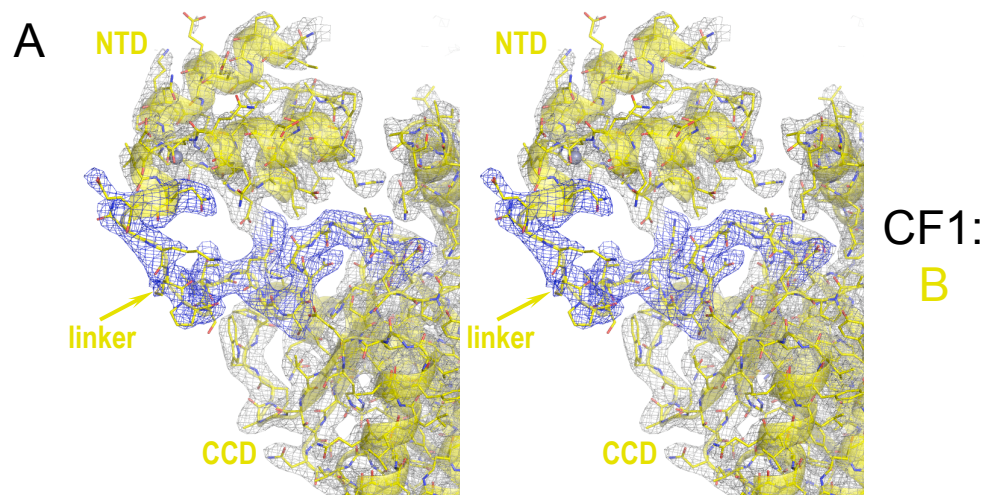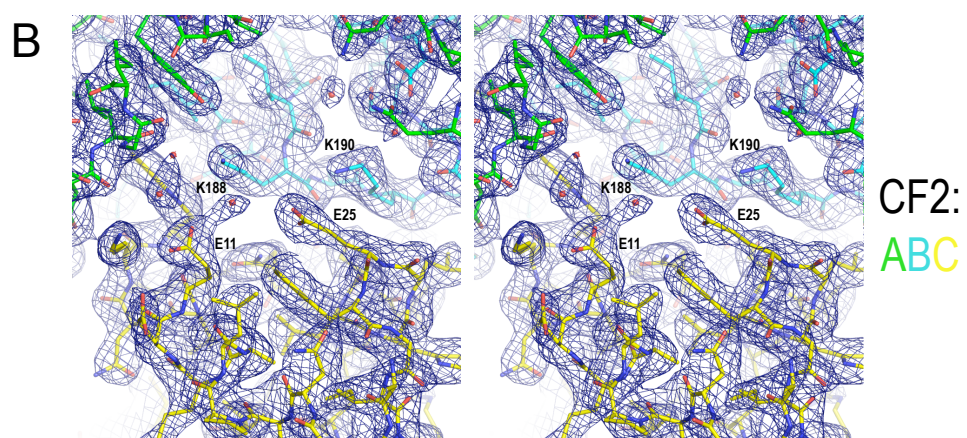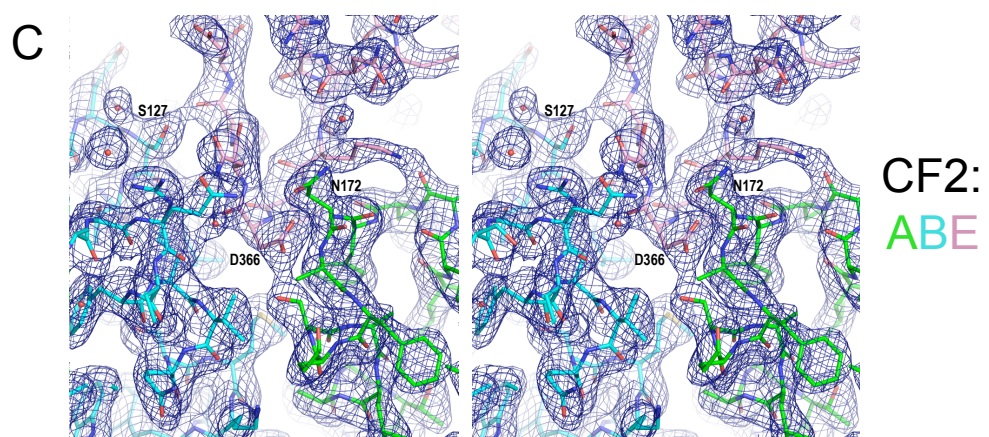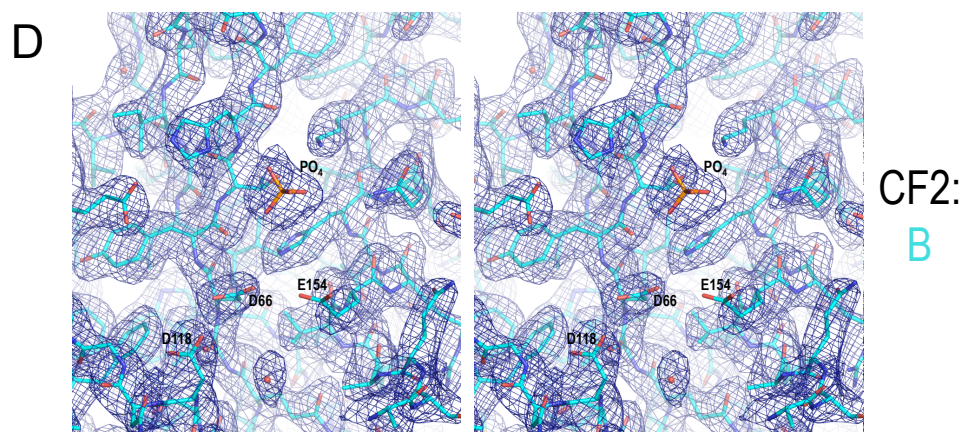

Supplement: Figure S5 — Examples of weighted 2Fo-Fc electron density maps for the refined structures. (A) IN chain B in CF1. Electron density, displayed as chicken wire, is colored blue for the NTD-CCD linker region (residues 44–61) and gray for the rest of the chain. The protein is shown as sticks and semitransparent cartoon. The NTD, CCD and linker are indicated. (B) The interface involving chain C NTD and the AB CCD dimer in CF2. (C) The interface of LEDGF chain E with AB CCD in CF2. (D) Active site of IN chain B with an associated phosphate ion in CF2. Note that a phosphate ion has been observed in a structurally identical position in two HIV-1 IN structures (PDB IDs 1k6y and 2b4j). The map in panel A is contoured at 1σ and those in panels B–D at 1.2σ. Carbon atoms are colored by chain as indicated in the legends to the right, and other atoms are colored blue for nitrogen, red for oxygen, yellow for sulfur, or orange for phosphorus. The gray sphere is zinc; red spheres are water molecules. (9.87 MB PDF) [file ppat.1000515.s005.pdf]
